# Facial mucocele and brain complications: diagnostic priorities

**DOI:** 10.1259/bjrcr.20190006

**Published:** 2019-11-15

**Authors:** Rebeca Sigüenza González, Santiago Pérez Fernández, Isabel Jiménez Cuenca, Carlos Castañeda Cruz

**Affiliations:** 1 Department of Radiology, Clinical Hospital, Valladolid, Spain

## Abstract

We present the case of a male with a history of nasal polyposis underwent bilateral nasosinusal endoscopic surgery. He went to the emergency department because of having behavioral changes and left frontal headache. An emergency CT showed nasal-sinus polyposis and several nodular lesions with a characteristic “ring” enhancement and perilesional edema. These findings were compatible facial mucocele complicated with rupture of the bone wall of the left frontal sinus and frontal abscess. Urgent surgery was performed, with left frontal craniectomy and drainage of the abscesses. Mucoceles are benign slowly growing lesions which can associate important complications. The most frequent are abscesses and the invasion of neighboring structures. It is very important to remember that frontal mucoceles can cause intracranial invasion when there is an erosion of the internal osseous table.

## Clinical presentation

A 79-year-old male with a history of nasal polyposis underwent bilateral nasosinusal endoscopic surgery.

He went to the emergency department because of having behavioral changes from 20 days ago and left frontal headache for 1 month. Blood test showed mild leukocytosis without associated elevation of acute phase reactants. Urgent CT was requested with the aim of ruling out brain complications.

## Differential diagnosis

There are multiple entities that can present characteristics similar to mucoceles. It is necessary to consider the following entities within their differential diagnosis: mucous retention cyst, inflammatory polyp, mycetoma, and the neopasias of the paranasal sinuses.

## Imaging findings

**Figure 1.**
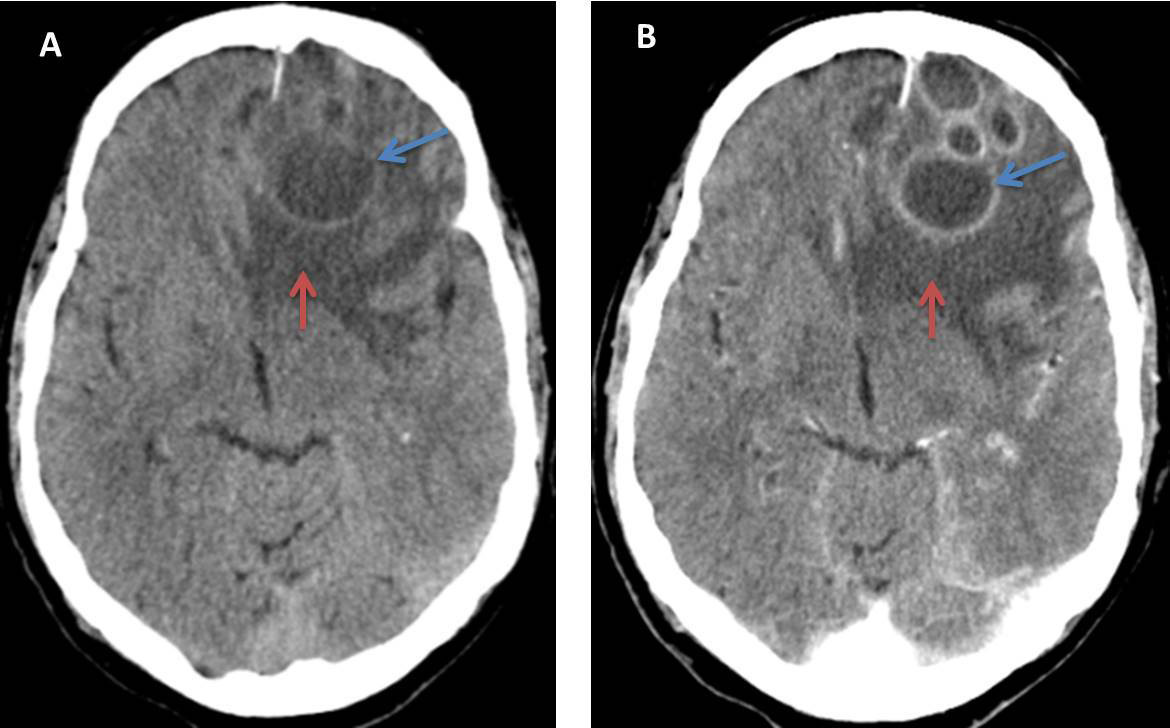
Axial scans cerebral CT without intravenous contrast (A) and after administration of intravenous contrast (B). Several frontal left hypodense nodular lesions (red arrow in A) with “ring” enhancement (red arrow in B) are visualized. They associate perilesional edema (blue arrows in A, B).

**Figure 2.**
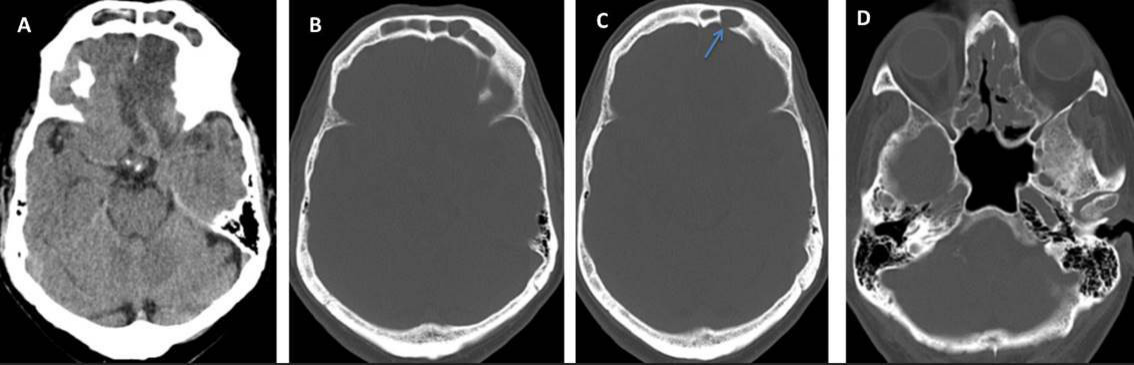
Axial scan cerebral CT in A and B (bone window) shows occupation of both frontal sinuses by soft tissue, which cause erosion of its left posterior wall (arrow in C). These features are compatible with frontal mucocele. In addition, polyposis at bilateral ethmoid sinus was associated.

CT showed several nodular lesions with a characteristic “ring” enhancement, accompanied by marked perilesional edema which caused mass effect (Figure 1). In addition, associated signs of nasal-sinus polyposis affecting the anterior and posterior ethmoidal cells were identified. Other important feature was the complete occupation of both frontal sinuses, with a bone defect in the posterior wall of the left frontal sinus (Figure 2). These findings were compatible facial mucocele complicated with rupture of the bone wall of the left frontal sinus and frontal abscess.

## Treatment

Urgent surgery was performed, with left frontal craniectomy and drainage of the referred abscesses. Samples were taken for microbiological cultures, with result of positive *Staphylococcus* *aureus*. The post-surgical evolution was favorable and the patient was discharged 10 days later.

### Outcome, follow up and discussion

Mucoceles are benign, slow-growing cystic lesions filled with mucus and lined with epithelium. They usually associate occupation and expansion of the paranasal sinus in which they originate. The formation of mucoceles in the paranasal sinuses is associated with obstruction of the sinus drainage ostium.

As a general rule, all those pathologies that hinder or prevent sinus drainage have the potential to produce mucoceles. The entities most associated with their training are the inflammatory–infectious processes, the trauma and the surgery, being less frequent its association with fibro-osseous lesions of the facial mass.

The most frequent location of the mucoceles are the frontal sinuses (60–67%), followed by the ethmoidal cells (20–25%), maxillary sinuses (5–10%) and sphenoid sinuses (<5%).^1^ They can be located simultaneously in more than one sinus region or even be bilateral, although the latter happens rarely.^1^

Over time, the mucoid material retained inside the corresponding paranasal sinus causes an increase in sinus pressure and its bony walls turn thinner. Secondary, bone osteolysis and remodeling are produced. In advanced cases, the lesion may reach the locoregional structures (orbit and/or brain parenchyma). It may cause intracranial complications of infectious etiology.

Imaging tests play a fundamental role in its diagnosis, mainly CT and MRI. In CT, mucoceles usually have a homogeneous content of low attenuation However, the density may vary depending on the content of water and proteins, as with the intensity of signal in the MR. In latter stages, mucoceles with a high concentration of water will be hyperintense in *T1*_1_ weighted sequences and hypointense in *T2*. As the protein content increases, the signal will increase in the *T1* weighted images and will decrease in *T2*.^2^

Conventional CT and MRI provide anatomic information and rely on morphologic changes secondary to paranasal lesions or brain tumors. However, they provide very little information about tumor functional behavior.^3^ Nowadays, new techniques like perfusion CT (PCT) and functional MRI modalities let to study tumor physiology.

On the one hand, the most important advantage of PCT is the linear relationship between contrast concentration and attenuation in CT, it facilitates quantitative measurement of blood flow and volume flow, so it let to identify tumor angiogenesis.^4,5^ On the other hand, functional MRI modalities such as diffusion MRI, dynamic susceptibility perfusion contrast MRI and MR spectroscopy also have been applied to investigate paranasal/brain tumors.^6^ Furthermore, diffusion has been used to differentiate benign and malignant lesions, due to the apparent diffusion coefficient (ADC) values of tissues vary according to their patology and cellularity.^7^

In spite of being benign diseases, the mucoceles have complications, being the most frequent the superinfection and the invasion by contiguity of neighboring structures. Therefore, the knowledge of its main manifestations, both clinical and radiological, will be crucial to reach an early diagnosis that allows guiding the therapeutic attitude, avoiding that the prognosis of a benign entity is overshadowed by the appearance of such complications.

The clinical case that concerns us represents a combination of both entities: the invasion by contiguity of neighboring structures such as brain parenchyma and superinfection/abscess. The structures that are affected by neighborhood depend on the location of the sinus in which the mucocele originates. On the one hand, orbital invasion is more frequent in the ethmoid and frontal sinus. On the other hand, sphenoid mucoceles are more likely to invade the cavernous sinuses, carotid vessels, optic nerves and the anterior and/or medial cranial fossa. Special mention should be made of the frontal mucoceles, which due to their proximity to the anterior cerebral fossa can cause intracranial invasion. This fact occurs when there is an erosion of the internal osseous table, causing important intraparenchymal complications, most of them of an infectious nature: cerebral abscesses, epidural/subdural abscesses or meningitis. The case presented is a clear example of this assumption. In the context of a marked nasosinusal polyposis and longstanding frontal mucocele, a thinning and solution of continuity of the posterior bony wall of the left frontal sinus was developed, with the consequent opening of the mucoid material to the ipsilateral frontal lobe and subsequent superinfection.

The main objective of this work is to emphasize the importance of knowledge of the main ways of presenting these complications. It is vital to perform an imaging test that complements the clinical suspicion, mainly CT. It is very important to remember diagnosis keys such as the “ring” enhancement (Figure 1) or the air bubbles which are seen inside the superinfected lesions.^6^ In addition, when a CT is performed, it is very important to pay attention to the bone window (Figure 2, arrow A) to identify possible erosions in the sinus walls that translate the intracranial extension of the lesion.

## Learning points

- Mucoceles are benign slowly growing lesions which can associate important complications.
- The most frequent location of the mucoceles is the frontal sinus.
- Imaging tests play a fundamental role in its diagnosis, mainly CT and MRI.
- Mucoceles have complications, being the most frequent the superinfection and the invasion of neighboring structures.
- Frontal mucoceles can cause intracranial invasion when there is an erosion of the internal osseous table.
- Infectious intraparenchymal complications (epidural/subdural abscesses or meningitis) are seen, secondary to intracranial invasion.
- Bone erosions in the sinus walls translate intracranial extension of the lesion.
